# Detrainment of plumes from vertically distributed sources

**DOI:** 10.1007/s10652-016-9492-x

**Published:** 2016-11-22

**Authors:** Rachael Bonnebaigt, C. P. Caulfield, P. F. Linden

**Affiliations:** 1 Department of Applied Mathematics and Theoretical Physics, University of Cambridge, Wilberforce Rd, Cambridge, CB3 0WA UK; 2 BP Institute, University of Cambridge, Madingley Rise, Cambridge, CB3 0EZ UK

**Keywords:** Plumes, Detrainment, Buoyancy, Stratification

## Abstract

We present experimental results demonstrating that, for the turbulent plume from a buoyancy source that is vertically distributed over the full area of a wall, detrainment qualitatively changes the shape of the ambient buoyancy profile that develops in a sealed space. Theoretical models with one-way-entrainment predict stratifications that are qualitatively different from the stratifications measured in experiments. A peeling plume model, where density and vertical velocity vary linearly across the width of the plume, so that plume fluid “peels” off into the ambient at intermediate heights, more accurately captures the shape of the ambient buoyancy profiles measured in experiments than a conventional one-way-entrainment model does.

## Introduction

Vertically distributed buoyancy sources, for example, radiators and walls heated by the sun, are commonly found in buildings. The comfort of occupants is affected by the temperature stratification that develops in the building. Different buoyancy sources drive different flows and result in different temperature stratifications. Buoyancy sources usually drive turbulent plumes, which can be simply described using the Morton et al. [12] plume model. There are three key assumptions in this model:

1. Profiles of density and vertical velocity across the plume are self-similar with height, and for simplicity, top hat profiles (i.e. constant and non-zero within the plume, zero outside) are assumed.
2. Ambient fluid is entrained into the plume at a rate proportional, via an entrainment coefficient *α*, to the characteristic vertical velocity at that height.
3. Changes in density are small compared with a reference density (the Boussinesq approximation).

This plume model has been applied, by many authors, to a variety of situations in the built environment. See, for example, [9].

The plume model may be applied to a filling box with either a point source or a horizontal line source. In a filling box, the space is sealed, and there is an initial transient as the buoyant plume rises to the ceiling, where it spreads out, forming a stratified region, which grows deeper in time. The interface between this stratified region and the initial ambient fluid is called the first front. Baines and Turner [2] find an expression for the position of the first front with time, and use their experimental data to find a value of $\alpha =0.10$ for the entrainment coefficient. Worster and Huppert [13] consider the time-dependent ambient buoyancy profile, and find good agreement between the numerical solution of their governing equations and their approximate analytic expression for the ambient buoyancy profile. In Sect. 3.1, we discuss this horizontal line source model, and compare a series of experiments with the theoretical model. Cooper and Hunt [5] present a model, which we discuss in Sect. 3.2, for a filling box containing a vertically distributed (over the full height of a wall) buoyancy source. We refer to this source as a full wall source. In a ventilated room with a full wall source, the plume fluid may reach its neutral buoyancy height at an intermediate height and spread horizontally in the room. Cooper and Hunt [5] find that, if the plume is assumed uniform across its width, meaning that the intrusion occurs at a single height, the resulting ambient stratification is unstable with respect to small perturbations in the plume flow or the ambient stratification. To solve this problem, they assume that the plume has a linear buoyancy profile across its width. However, in the unventilated case, they neglect this variation in buoyancy profile across the width of the plume, and so, in their model for the unventilated room, the plume can never detrain. Caudwell et al. [3] also consider a vertically distributed source, but their source is held at a constant temperature, rather than providing a constant flux. In their experiments, the plume remained laminar for some distance, leading them to develop a hybrid model, combining a laminar part with a Cooper-and-Hunt-like turbulent part. Whilst this hybrid model better described the ambient buoyancy profile at small heights, it failed to capture the shape of the profile at the top of the space.

Cooper and Hunt [5], Linden et al. [10], and Chen et al. [4] all consider a ventilated room with a vertically distributed, constant flux source. In this case, the plume can reach its neutral buoyancy height at an intermediate height within the room, where it intrudes into the ambient. Linden et al. [10] assume that the intrusion depth is negligible, with the result that the model predicts layers of different density in the ambient, although in their experiments these layers were not seen. Cooper and Hunt [5] show that this layered stratification is unstable to small perturbations in plume flow or ambient stratification, thus it is not expected to be physically realised. Instead, they allow intrusions to have a finite depth. Chen et al. [4] also observe intrusions in experiments with a vertically distributed buoyancy source in a naturally ventilated space. They suggest that these intrusions smooth out the layered profile predicted by Linden et al. [10]. Both Cooper and Hunt [5] and Linden et al. [10] predict intrusions only for a ventilated space. In particular, they do not allow for detrainment of the plume in a unventilated space. Importantly, this neglect of detrainment is made, despite Cooper and Hunt [5] proposing a linear buoyancy profile across the width of the plume for the ventilated space, which in principle actually does allow detrainment in both ventilated and unventilated spaces.

Detrainment, however, may be important for vertically distributed sources. In experiments with a vertical line source, Gladstone and Woods [7] observe detrainment: plume fluid intruding into the ambient at intermediate heights. If, rather than being entrained into a plume and flushed quickly out of the room, contaminants are repeatedly detrained from and entrained into a plume, air quality may be affected. In experiments with a vertical ice wall as a source, McConnochie and Kerr [11] find that, in the stratified region below the first front, ambient buoyancy profiles are approximately linear. They suggest that this disagreement between the profiles from their experiments and the profiles predicted by the models of Cooper and Hunt [5] and Linden et al. [10] is due to detrainment. When detrainment has a significant effect, a peeling plume model, such as that of Hogg et al. [8] may be appropriate—here, density and vertical velocity are assumed to vary linearly across the plume, allowing parts of the plume to “peel” off into the ambient as outer parts of the plume reach their neutral buoyancy height at intermediate heights. We extend this peeling plume model, applying it to a vertically distributed buoyancy source, and find that the peeling plume model captures the shape of the ambient buoyancy profile more accurately than the model of Cooper and Hunt [5].

We consider two sources in a sealed space: a horizontal line source and a full wall source. We wish to know whether they have one-way-entrainment, as is conventionally assumed, or whether they also have detrainment. First, in Sect. 2, we present the results of experiments with each of the two sources. Then, in Sect. 3, we present theoretical models for each source, which we compare with our experimental results. We compare the measurements of the buoyancy profiles produced by a line source with the model of Worster and Huppert [13] in Sect. 3.1. With the full wall source, Sect. 3.2 shows that our experimental results disagree with the Cooper and Hunt [5] model, so we compare our experimental results with a peeling plume model. Finally, Sect. 4 contains the conclusions of this work.

## Experiments

### Methods

**Fig. 1:**
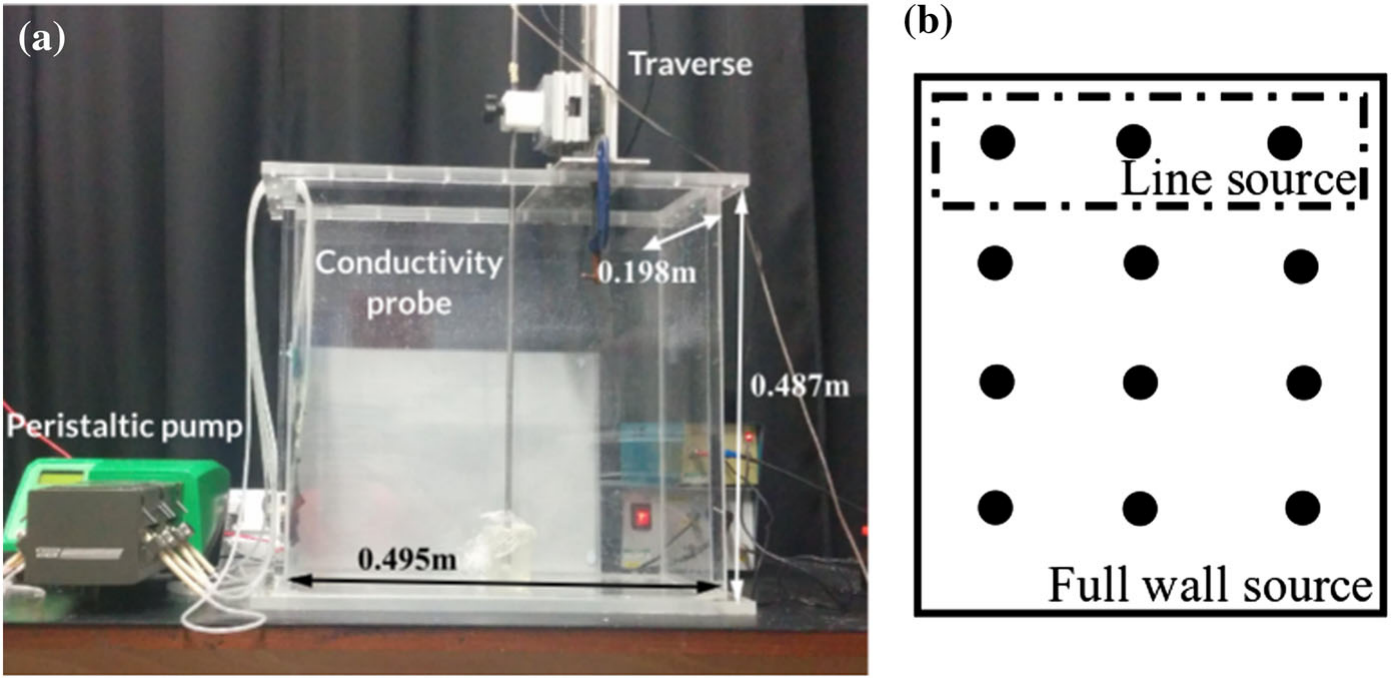
The experimental setup (**a**), *left*, with a schematic showing details of the source (**b**), *right*. Individual source tubes are indicated by *circles*. The three topmost source tubes, enclosed by the *dashed box*, form the line source, while, for the wall source, all of the source tubes are used

We perform experiments with each of the sources, to investigate whether detrainment occurs. In these experiments, salt provides the density differences. Figure 1 shows the experimental setup: the 0.487 m tall tank is filled to approximately 0.3 m with fresh water. A source (described below) covers one wall of the tank, and this wall is 0.198 m wide. The other wall is 0.495 m long.

To approximate a line source, salt water is pumped through three tubes, which are distributed (in a line) over the width of the tank wall. The spacing of these sources is kept the same in each experiment. Figure 1b shows the source setup for the full wall source of Sect. 2.3; using just the top row of tubes, shown by the dashed box, gives a line source. The end of each tube is covered with a fine mesh fabric to ensure that the fluid leaving the source is turbulent. Two Watson Marlow 520Du peristaltic pumps with 505L and 505LX pumpheads pump salt water through the tubes. A total of 6 tubes run through the pumpheads and are then split into two, giving 12 tubes which make up the full wall source. The spacing between these tubes is kept the same in each experiment, and was chosen so that the sources were evenly distributed across the wall.

A conductivity probe, traversed vertically through the tank over a height of 0.25 m every 2 min, measures the stratification. The conductivity probe is calibrated using a range of samples of salt water whose densities are measured using a density meter. The measured stratification is used to calculate the dimensionless ambient buoyancy, defined as $\alpha ^{2/3}f_0^{-2/3}H g \frac{\left( \rho _a-\rho _1 \right) }{\rho _1}$ for the line source, where $\alpha$ is the entrainment coefficient, $f_0$ is the source buoyancy flux per unit source width, *H* is the tank height, *g* is gravitational acceleration, $\rho _1$ is the initial ambient density, and $\rho _a$ is the ambient density. For the point source, Worster and Huppert [13] define the dimensionless ambient buoyancy as $4\pi ^{2/3}\alpha ^{4/3}f_0^{-2/3}H^{5/3}g \frac{\left( \rho _a-\rho _1\right) }{\rho _1}$ (using our notation). The differences between the two nondimensionalisations are due to the fact that Worster and Huppert [13] considered a point source, whereas we consider a line source. For the full wall source, $f_0$ is replaced by $b_{s_0}H$, where $b_{s_0}$ is the source buoyancy flux per unit area. The resulting experimental ambient buoyancy profiles, such as those presented below in Fig. 3, show scatter of approximately 1 dimensionless ambient buoyancy unit, compared with a maximum dimensionless ambient buoyancy of about 2–9 (i.e. 11–50% scatter). The source fluid is dyed with food colouring, using different colours at different times during the experiment, to visualise the flow. When the plume detrains, we observe dyed fluid exiting the plume and entering the ambient at an intermediate height.

### Horizontal line source

**Fig. 2:**
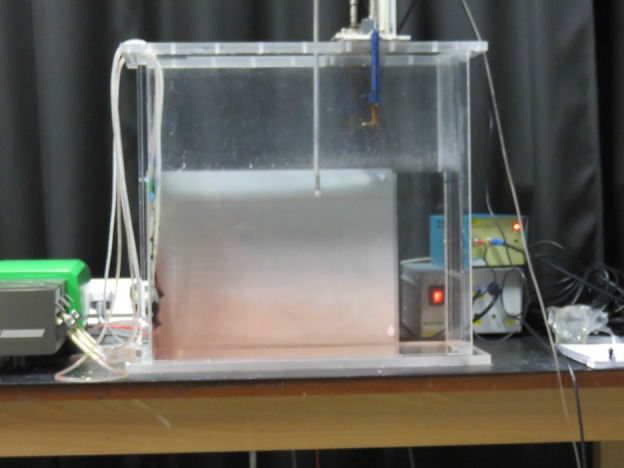
Dyed plume fluid in the line source experiments is not observed to detrain. Instead, it descends to the *bottom* of the tank. The photo shows experiment $T_{\text {line}}$ at 8 min after the start of the experiment. The source fluid was dyed red at 6 min into the experiment

**Fig. 3:**
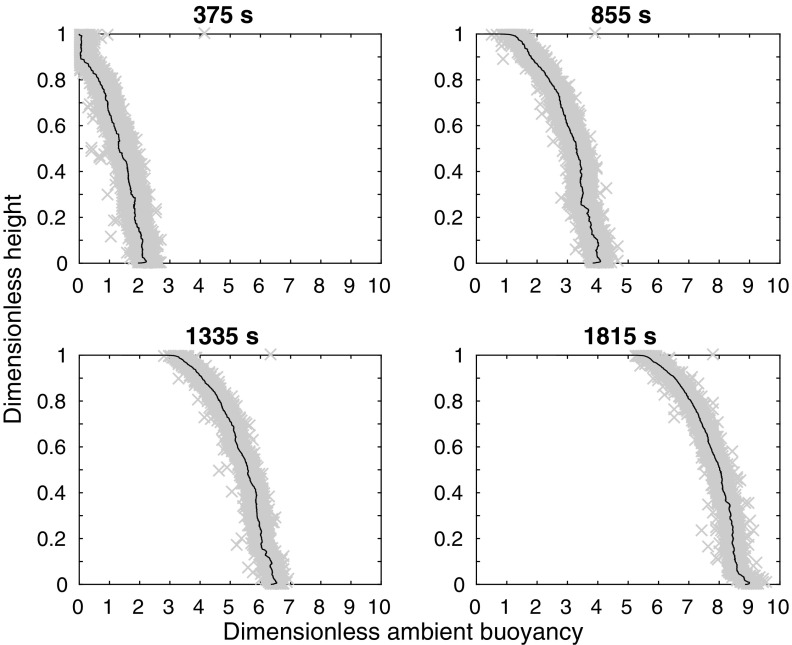
Dimensionless ambient buoyancy profiles for line source experiment ${\text {N}}_{\text {line}}$ (which has a flow rate of $0.9{\text {ml}/\text {s}}$ and a dimensionless density difference $\Delta \rho =0.13$). The four subplots show the experimental results at four different times: 375, 855, 1335, and 1815 s after starting. When nondimensionalised by the timescale $W\alpha ^{-2/3} f_0^{-1/3}$, where $\alpha$ is the entrainment coefficient, $f_0$ is the source buoyancy flux per unit source width, and *W* is the tank width, these times are 1.6, 3.7, 5.7, and 7.8.* Grey crosses* show the original data and a *black line* shows the filtered data (filtered using a median filter)

We performed 20 experiments with a line source, varying both the source density and the source volume flux; these experiments are listed in Table 1. Whilst the dimensionless density differences $\Delta \rho =\left( \rho _a-\rho _1\right) /\rho _1$ between the source and the initial ambient for experiments $E_{\text {line}},J_{\text {line}},O_{\text {line}},$ and $T_{\text {line}}$ are large compared with those for experiments $A_{\text {line}},F_{\text {line}},K_{\text {line}},$ and $P_{\text {line}}$, entrainment rapidly reduces the density, and so the Boussinesq approximation works well soon after the fluid leaves the source. The density profiles from a typical experiment ($N_{\text {line}}$, which, as listed in Table 1, has a flow rate of $0.9{\text {ml}}/{\text {s}}$ and a dimensionless density difference $\Delta \rho =0.13$) at four different times (375, 855, 1335, and 1815 s after starting) are shown in Fig. 3. In each of the profiles shown, near the bottom of the tank (dimensionless height zero), the density gradient is very small. In all of these line source experiments we observed this dye descending to the floor of the tank as part of the plume, as shown in Fig. 2, rather than intruding into the ambient, so we believe that detrainment did not occur. In Sect. 3.1, we will compare these experimental results with the one-way-entrainment model of Worster and Huppert [13], showing that our experimental results agree with the theory, and so our method of producing a line source (having several individual sources) is appropriate.

**Table 1:** The dimensionless density difference $\Delta \rho$ between the source and the initial ambient for the 20 line source experiments, which have the source buoyancy fluxes listed in Table 2

Flow rate (ml/s)	Approximate amount, by volume, of saturated salt water				
	20%	40%	60%	80%	100%
0.4	${\text {A}}_{\text {line}}$, $\Delta \rho =0.04$	${\text {B}}_{\text {line}}$, $\Delta \rho =0.04$	${\text {C}}_{\text {line}}$, $\Delta \rho =0.07$	${\text {D}}_{\text {line}}$, $\Delta \rho =0.10$	${\text {E}}_{\text {line}}$, $\Delta \rho =0.12$
0.6	${\text {F}}_{\text {line}}$, $\Delta \rho =0.02$	${\text {G}}_{\text {line}}$, $\Delta \rho =0.06$	${\text {H}}_{\text {line}}$, $\Delta \rho =0.09$	${\text {I}}_{\text {line}}$, $\Delta \rho =0.12$	${\text {J}}_{\text {line}}$, $\Delta \rho =0.18$
0.9	${\text {K}}_{\text {line}}$, $\Delta \rho =0.02$	${\text {L}}_{\text {line}}$, $\Delta \rho =0.05$	${\text {M}}_{\text {line}}$, $\Delta \rho =0.10$	${\text {N}}_{\text {line}}$, $\Delta \rho =0.13$	${\text {O}}_{\text {line}}$, $\Delta \rho =0.17$
1.1	${\text {P}}_{\text {line}}$, $\Delta \rho =0.03$	${\text {Q}}_{\text {line}}$, $\Delta \rho =0.06$	${\text {R}}_{\text {line}}$, $\Delta \rho =0.10$	${\text {S}}_{\text {line}}$, $\Delta \rho =0.14$	${\text {T}}_{\text {line}}$, $\Delta \rho =0.18$

**Table 2:** The source buoyancy flux per unit source width (line source) or source area (full wall source), $g \Delta \rho$, multiplied by the volume flux (in ${\mathrm {m}}^3{\mathrm {s}}^{-1}$), divided by the source width (line source) or source area (full wall source)

Experiment	Source buoyancy flux	
	Line source ($\times 10^{-6}{\mathrm {m}}^3{\mathrm {s}}^{-3}$)	Full wall source ($\times 10^{-6}{\mathrm {m^2}}{\mathrm {s}}^{-3}$)
*A*	0.8	6.3
*B*	0.8	12.7
*C*	1.4	19.0
*D*	2.0	25.3
*E*	2.4	34.8
*F*	0.6	10.7
*G*	1.8	21.4
*H*	2.7	32.1
*I*	3.6	53.5
*J*	5.3	64.1
*K*	0.9	15.0
*L*	2.2	37.6
*M*	4.5	52.7
*N*	5.8	75.2
*O*	7.6	90.3
*P*	1.6	19.0
*Q*	3.3	47.5
*R*	5.4	76.0
*S*	7.6	95.0
*T*	9.8	133.0

### Full wall source

**Fig. 4:**
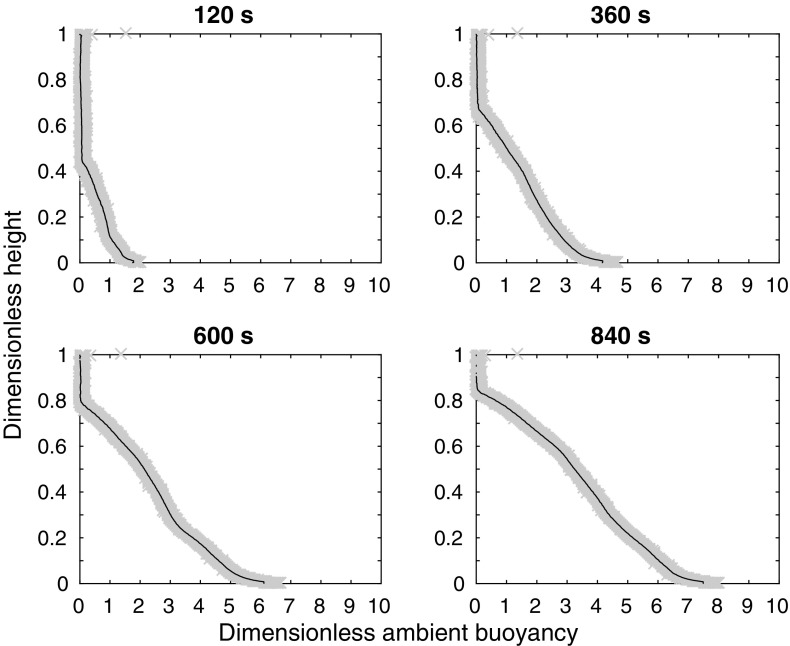
Dimensionless ambient buoyancy profiles for full wall source experiment ${\text {N}}_{\text {full}}$ (which has a flow rate of $3.8{\text {ml}}/\text {s}$ and a dimensionless density difference $\Delta \rho =0.10$). The four subplots show the experimental results at four different times: 120, 360, 600, and 840 s after starting. When nondimensionalised by the timescale $W\alpha ^{-2/3} b_{s_0}^{-1/3}H^{-1/3}$, where $\alpha$ is the entrainment coefficient, $b_{s_0}$ is the source buoyancy flux per unit source area, and *W* is the tank width, these times are 0.4, 1.3, 2.2, and 3.1.* Grey crosses* show the original data and a *black line* shows the filtered data (filtered using a median filter)

The experiments are set up as in Sect. 2.2, but using the full wall source, shown by the solid line box in Fig. 1b. We performed 20 experiments, varying both the source density and volume flux; these experiments are listed in Table 3. The density profiles from a typical experiment ($N_{\text {full}}$, which has a flow rate of 3.8 ml/s and a dimensionless density difference $\Delta \rho =0.10$) at four different times (120, 360, 600, and 840 s after starting) are shown in Fig. 4. Note that the source buoyancy fluxes in the line source experiments shown in Fig. 3 are not identical to those in the wall source experiments shown in Fig. 4, so the total buoyancy at each time is slightly different in the two figures. These profiles are qualitatively different from those in Fig. 3, which was for a horizontal line source—the full wall source profiles, rather than being almost vertical near the bottom of the tank, have a different slope and a change of curvature near the bottom of the tank. This difference is seen even before the first front reaches the top of the tank in the line source experiment (see the profile at 375 s shown in Fig. 3). Therefore, the difference is not only due to the fact that the profiles evolve at different rates, as there is also a qualitative difference between the shape of the profiles associated with a line source and the shape of the profiles associated with a wall source.

**Table 3:** The 20 full wall source experiments, including the dimensionless density difference $\Delta \rho$ between the source fluid and the initial ambient fluid

Flow rate ( ml/s)	Approximate amount, by volume, of saturated salt water				
	20%	40%	60%	80%	100%
1.6	${\text {A}}_{\text {full}}$, $\Delta \rho =0.02$	${\text {B}}_{\text {full}}$, $\Delta \rho =0.04$	${\text {C}}_{\text {full}}$, $\Delta \rho =0.06$	${\text {D}}_{\text {full}}$, $\Delta \rho =0.08$	${\text {E}}_{\text {full}},\,$ $\Delta \rho =0.11$
2.7	${\text {F}}_{\text {full}}$, $\Delta \rho =0.02$	${\text {G}}_{\text {full}}$, $\Delta \rho =0.04$	${\text {H}}_{\text {full}}$, $\Delta \rho =0.06$	${\text {I}}_{\text {full}}$, $\Delta \rho =0.10$	${\text {J}}_{\text {full}}$, $\Delta \rho =0.12$
3.8	${\text {K}}_{\text {full}}$, $\Delta \rho =0.02$	${\text {L}}_{\text {full}}$, $\Delta \rho =0.05$	${\text {M}}_{\text {full}}$, $\Delta \rho =0.07$	${\text {N}}_{\text {full}}$, $\Delta \rho =0.10$	${\text {O}}_{\text {full}}$, $\Delta \rho =0.12$
4.8	${\text {P}}_{\text {full}}$, $\Delta \rho =0.02$	${\text {Q}}_{\text {full}}$, $\Delta \rho =0.05$	${\text {R}}_{\text {full}}$, $\Delta \rho =0.08$	${\text {S}}_{\text {full}}$, $\Delta \rho =0.10$	${\text {T}}_{\text {full}}$, $\Delta \rho =0.14$

**Fig. 5:**
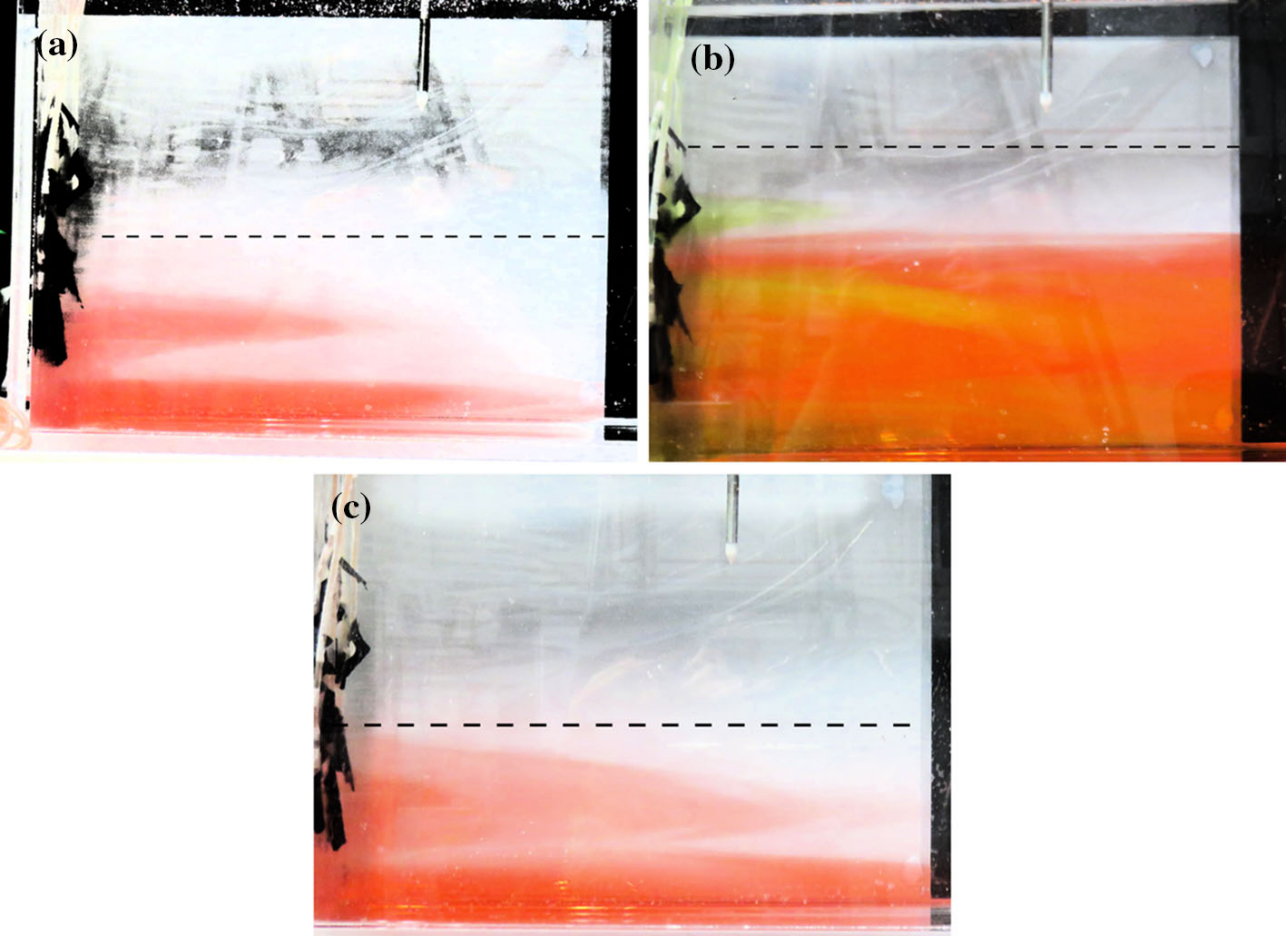
*Top left* **a** source fluid (*dyed red* 3 min into the experiment) detrains, intruding into the ambient, shown at 4 min into experiment ${\text {T}}_{\text {full}}$. *Top right* **b** source fluid (*dyed green* 7 min into the experiment) detrains, shown at 10 min into experiment $T_{\text {full}}$. Source fluid (*dyed red* 3 min into the experiment) detrains at similar heights towards the bottom of the tank for each experiment. *Bottom* **c** experiment $C_{\text {full}}$ at 6 min detrains at similar heights as experiment $T_{\text {full}}$, shown on the *top left*, at 4 min. The contrast has been enhanced in each photograph, for clarity. The *dashed lines* show the approximate height of the first front, separating the stratified region below it from the initial ambient fluid above

One reason for this difference in profile shape is, as in the vertical line source experiments of Gladstone and Woods [7], detrainment occurs. This detrainment is shown in Fig. 5, where the top left hand figure (a) shows red dyed source fluid intruding into the undyed ambient at intermediate heights, and the top right hand figure (b) shows green dyed source fluid intruding into the red (at lower heights) and undyed (at larger heights) ambient at intermediate heights. This occurs in each of the full wall source experiments. The fluid intrudes into the ambient over a range of heights, with the bottom part of this range being the same in each experiment. An example of this is shown in Fig. 5 which shows, on the top left (a), experiment $T_{\text {full}}$ at 4 min into the experiment, and, on the bottom row (c), experiment $C_{\text {full}}$ at 6 min into the experiment. Both experiments have intrusions over approximately the same range of heights. Over time, the intrusions extend horizontally, further into the ambient, and occur over a wider range of heights. Figure 5 shows this happening in experiment $T_{\text {full}}$: the top left hand figure (a) is at 4 min into the experiment, and has (red) intrusions over approximately the bottom third of the height to which the tank was filled, whilst the top right hand figure (c) is at 10 min into the experiment and has (green) intrusions over approximately the bottom two thirds of the height to which the tank was filled. Detrainment suggests that a one-way-entrainment model will be unable to predict the density profiles observed in experiments, and, instead, a peeling plume model is needed. We compare the experimental results with both the one-way-entrainment model and a peeling plume model in Sect. 3.2.

## Theoretical models

### Horizontal line source

To confirm that our line source, which is made up of several discrete sources, does indeed approximate a distributed source, we compare our line source experiments with the well-established Worster and Huppert [13] one-way-entrainment line source model. They use the Morton et al. [12] plume model to describe an axisymmetric plume in a sealed, insulated room. For completeness, we present (changing notation) the horizontal line source version of this model.

The volume, momentum, and buoyancy fluxes per unit length through the plume are defined as

1 $$q=\int _0^{\infty }w \, dx, \quad m= \int _0^{\infty } w^2 \, dx, \quad \text {and} \quad \textit{f}=\int _0^{\infty }wg \left( \frac{\rho _a - \rho }{\rho _1}\right) \, dx,$$

where *w* is vertical velocity, *x* is distance (from the wall) across the plume, *g* is acceleration due to gravity, $\rho _a$ is the ambient density, $\rho$ is the plume density, and $\rho _1$ is the initial ambient density. Conservation of volume, momentum, and buoyancy fluxes give the plume equations:

2 $$\frac{dq}{dz}= \alpha \frac{m}{q}, \quad \frac{dm}{dz}=\frac{qf}{m}, \quad {\text {and}} \quad \frac{df}{dz}=-q\frac{\partial \delta _a}{\partial z},$$

where *z* is vertical distance from the source, $\alpha$ is the entrainment coefficient, and $\delta _a=g\left( \rho _1-\rho _a\right) /\rho _1$ is the ambient buoyancy. The boundary conditions are

3 $$q=m=0 \quad \text {at} \quad \textit{z}=0, \quad \text {and} \quad \textit{f}=\textit{f}_0 \quad \text {at} \quad \textit{z}=0,$$

where $f_0$ is the source buoyancy flux per unit width. These boundary conditions are for a pure plume, and to account for the non-zero volume flux at the source, a virtual origin adjustment is required (as discussed by [12]). This adjustment assumes that the theoretical source is some distance above the experimental source, so that, at the height of the experimental source, the plume has a non-zero volume flux. For our line source experiments, the adjustment is small (a dimensionless value of about 0.008), however, so we neglect it for the full wall model.

Changes in ambient buoyancy are given by

4 $$\frac{\partial \delta _a}{\partial t} = \frac{q}{W} \frac{\partial \delta _a}{\partial z},$$

where *W* is the width of the room outside the plume (we assume that the plume is thin) and *t* is time. This assumes that the aspect ratio of the box, *H*/*W*, is small, and that the time for the box to be filled with dense fluid is much greater than the time the plume takes to rise through the box.

We nondimensionalise, taking the height of the tank *H* as the natural length scale, and defining dimensionless variables

5 $$q=\alpha ^{2/3}f_0^{1/3}H\,Q, \quad m=\alpha ^{1/3}f_0^{2/3}H \, M, \quad f=f_0\, F, \quad \delta _a=\alpha ^{-2/3}f_0^{2/3}H^{-1}\, \Delta _a, \quad z=H\,Z, \quad {\text {and}} \quad \textit{t}=W\alpha ^{-2/3}f_0^{-1/3}\,T.$$

The particular value of $\alpha$ that we use for the line source is $\alpha =0.04$, which is discussed further in Sect. 3.1.1. On substituting these dimensionless variables into (2), we obtain the dimensionless plume equations

6 $$\frac{dQ}{dZ}=\frac{M}{Q}, \quad \frac{dM}{dZ}=\frac{QF}{M}, \quad {\text {and}} \quad \frac{dF}{dZ}=-Q\frac{\partial \Delta _a}{\partial Z},$$

and on substituting the dimensionless variables (5) into (4), we obtain the dimensionless ambient buoyancy equation

7 $$\frac{\partial \Delta _a}{\partial T}=Q\frac{\partial \Delta _a}{\partial Z}.$$

The boundary conditions (3) become

8 $$Q=M=0 \quad \text {at} \quad \textit{Z}=0, \quad \text {and} \quad \textit{F}=1 \quad \text {at} \quad \textit{Z}=0.$$

The timescale, $W \alpha ^{-2/3} f_{0}^{-1/3}$, is given in Table 4 for each of the 20 experiments performed, and varies between approximately 3 and 8 min.

**Table 4:** The timescales $W \alpha ^{-2/3} f_0^{-1/3}$ for the 20 line source experiments

Experiment	${\text {A}}_{\text {line}}$	${\text {B}}_{\text {line}}$	${\text {C}}_{\text {line}}$	${\text {D}}_{\text {line}}$	${\text {E}}_{\text {line}}$	${\text {F}}_{\text {line}}$	${\text {G}}_{\text {line}}$	${\text {H}}_{\text {line}}$	${\text {I}}_{\text {line}}$	${\text {J}}_{\text {line}}$
Timescale(s)	450	463	389	344	317	508	354	309	281	243

Experiment	${\text {K}}_{\text {line}}$	${\text {L}}_{\text {line}}$	${\text {M}}_{\text {line}}$	${\text {N}}_{\text {line}}$	${\text {O}}_{\text {line}}$	${\text {P}}_{\text {line}}$	${\text {Q}}_{\text {line}}$	${\text {R}}_{\text {line}}$	${\text {S}}_{\text {line}}$	${\text {T}}_{\text {line}}$
Timescale(s)	435	321	258	234	215	350	281	241	217	199

Equations (6) and (7), with boundary conditions (8), are solved numerically, using the method of [6]. This method assumes that, at $Z=0$, the plume lays down layers of dense fluid. By tracking the position and thickness of these layers with time, we obtain the ambient stratification.

#### Comparing the model with experiments

**Fig. 6:**
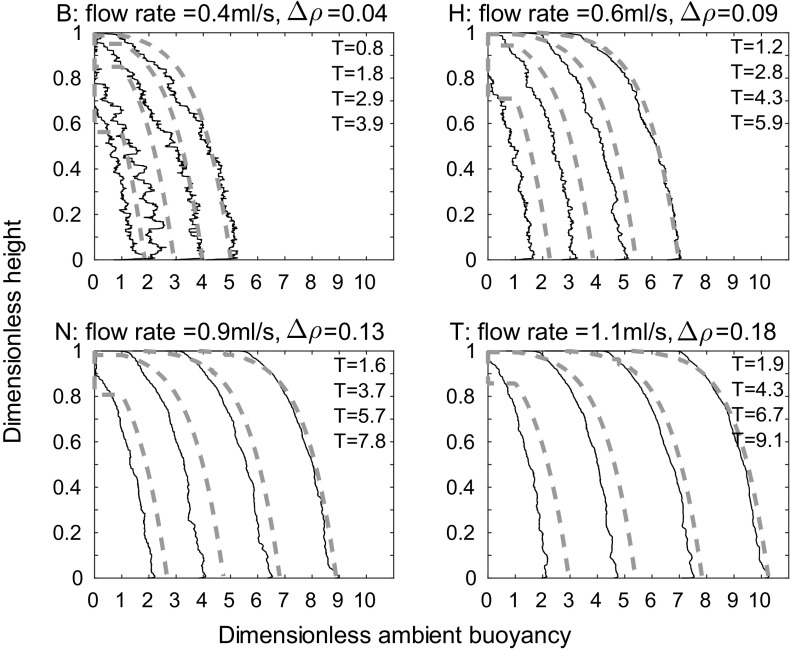
Dimensionless ambient buoyancy profiles for line source experiments ${\text {B}}_{\text {line}}$, ${\text {H}}_{\text {line}}$, ${\text {N}}_{\text {line}}$, and ${\text {T}}_{\text {line}}$. *Solid black lines* show filtered experimental results, the *dashed lines* show the model results. Each subplot shows profiles at times 375, 855, 1335, and 1815 s after starting, as in Fig. 3. The corresponding dimensionless times are listed on each plot

Figure 6 shows qualitative agreement between the theoretical model and our experimental results (representative experiments $B_{\text {line}}$, $H_{\text {line}}$, $N_{\text {line}}$, and $T_{\text {line}}$, which have different source buoyancy fluxes, as listed in Table 2, are shown). Each subplot shows the ambient buoyancy profile at times 375, 855, 1335, and 1815 s after starting. The time used for the model is the time at which the probe has traversed through half of the height of the tank. Conservation of buoyancy appears to improve with time. This apparent improvement occurs because the probe measures a profile at a single horizontal distance from the source. Since the dense fluid from the plume takes some time to spread as a gravity current across the bottom of the tank, there are horizontal inhomogeneities at early times which are not captured by the probe.

**Fig. 7:**
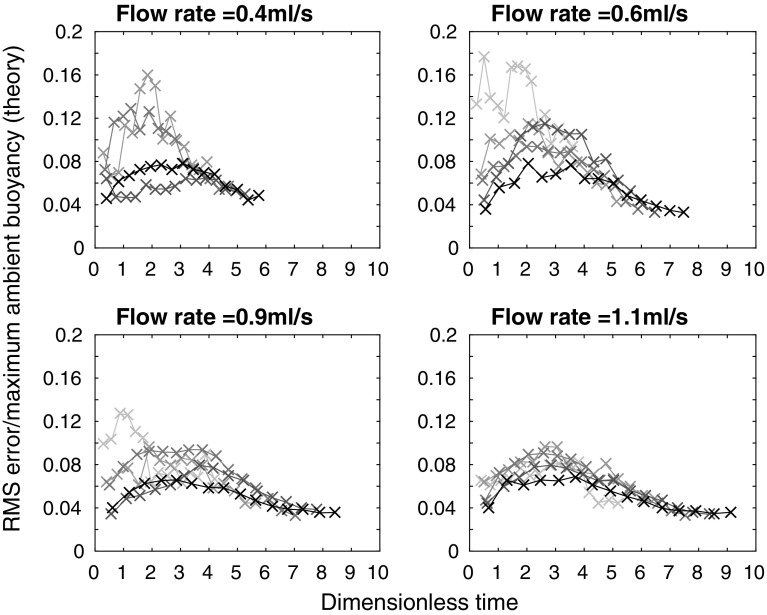
RMS error between theoretical and experimental dimensionless ambient buoyancy profiles, divided by maximum theoretical dimensionless ambient buoyancy, against dimensionless time for each of the 20 line source experiments listed in Table 1, apart from experiment A which has a large RMS error, and is not shown

A more quantitative measure of the agreement between the theoretical and experimental profiles is the root mean square (RMS) error, found by calculating, point by point, the square of the difference between the theoretical and experimental profiles, then taking the average over the tank height, and finally, taking the square root. The results of these calculations are shown in Fig. 7, where each subplot shows a different source flow rate. On each subplot, the five different lines correspond to the five different source densities used at that source flow rate, with darker lines for larger source densities. The RMS error was calculated at each time that a density profile was measured during an experiment—the crosses on each line correspond to these calculations. The error is smaller at larger times, in agreement with visual comparison of the profiles in Fig. 6. At all but the lowest source volume flux and density, in each experiment, the RMS error in dimensionless ambient buoyancy at late times is typically below 4% of the maximum theoretical dimensionless ambient buoyancy in that experiment. The RMS error is thus relatively small compared with the scatter in the original experimental data, and we conclude that the theoretical model provides a good description of the experimental profiles. In these experiments, the value of the entrainment coefficient that gives a low RMS value at late times (the RMS error does vary with $\alpha$) and a good visual fit between theoretical and experimental dimensionless ambient buoyancy profiles is $\alpha =0.04$. This value is lower than other works on wall-bounded line sources (e.g. $\alpha =0.073$ in [1]), but we expect our value to be lower because our source entrains only over part of the width of the tank, as it is made up of discrete sources.

**Fig. 8:**
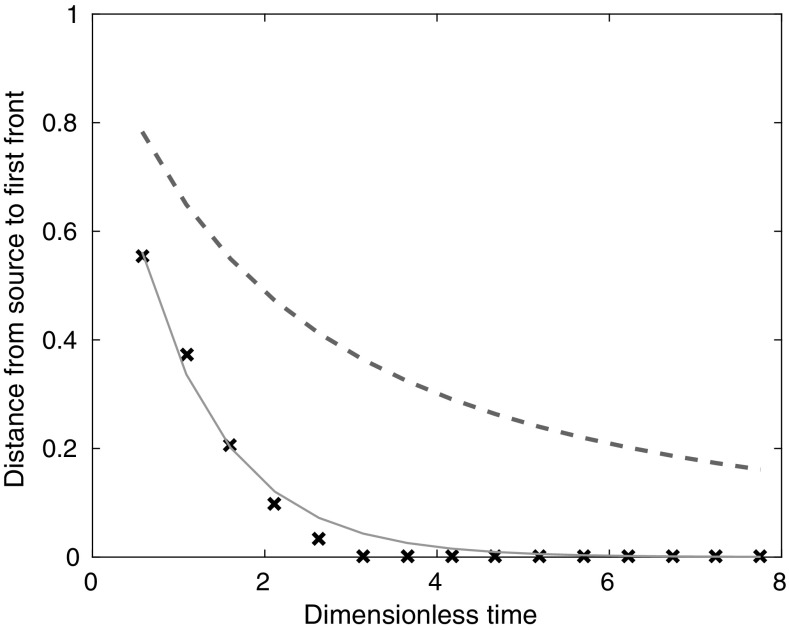
The distance from the source to the first front for experiment $N_{\text {line}}$ (crosses), from line source theory (*solid line*), and from half axisymmetric source theory (*dashed line*), with $\alpha =0.04$ for both models. (This value of $\alpha$ gives the best fit between the theoretical and experimental buoyancy profiles.) The line source theory and the half axisymmetric source theory predict very different first front speeds

To ensure that the three sources are, together, acting as a line source, rather than as three independent half axisymmetric plumes, we compare the experimental first front heights from a representative experiment, $N_{\text {line}}$, with the theoretical first front heights for a line source and a single axisymmetric source (from [13], adjusted to be for a half source) in Fig. 8. The crosses are the experimental results, the solid line is the line source theory, and the dashed line is the half axisymmetric source theory. The line source theory gives much better agreement with the experimental results than the axisymmetric source gives, and so we conclude that our discrete sources in a line do indeed act as a line source, confirming that our method of producing a distributed source is appropriate.

### Full wall source

Having confirmed that our method of producing a distributed source is appropriate, we now consider a full wall source. Cooper and Hunt [5] present a model, based on the Morton et al. [12] one-way-entrainment plume model, for a filling box with a full wall source. For completeness, we present their model here (changing notation) then, in Sect. 3.2.1, compare our experimental results with the model, showing it to be an inadequate model when detrainment occurs. As in Sect. 3.1, conservation of volume, momentum, and buoyancy fluxes give the plume equations. The volume and momentum flux equations are as in (2), but, in the buoyancy flux equation, an extra term, the source buoyancy flux per unit source area $b_s(z)$, accounts for the vertically distributed buoyancy source,

9 $$\frac{df}{dz}=-q \frac{\partial \delta _a}{\partial z} + b_s(z).$$

We nondimensionalise as in (5), replacing $f_0$ by $b_{s_0} H$, where $b_{s_0}=b_s(0)$ is the source buoyancy flux per unit area at $z=0$. The dimensionless plume equations with one-way-entrainment are then

10 $$\frac{dQ}{dZ}=\frac{M}{Q}, \quad \frac{dM}{dZ}=\frac{QF}{M}, \quad {\text {and}} \quad \frac{dF}{dZ}=-Q\frac{\partial \Delta _a}{\partial Z} + B(Z),$$

where $B(Z)=b_s(z)/b_{s_0}$ is the nondimensional source buoyancy flux per unit area. For the full wall source $B(Z)=1$. Boundary conditions are now

11 $$Q=M=F=0 \quad \text {at} \quad \textit{Z}=0.$$

Equation (7) remains the appropriate equation for the ambient buoyancy. The timescale, $W \alpha ^{-2/3} {b_s}_{0}^{-1/3} H^{-1/3}$, is given in Table 5 for each of the 20 experiments performed, and varies between approximately 4 and 11 min. Equations (10) and (7), with boundary conditions (11), are solved numerically, using the method of [6], as described in Sect. 3.1.

**Table 5:** The timescales $W \alpha ^{-2/3} {b_s}_{0}^{-1/3} H^{-1/3}$ for the full wall experiments, with $\alpha =0.018$

Experiment	${\text {A}}_{\text {full}}$	${\text {B}}_{\text {full}}$	${\text {C}}_{\text {full}}$	${\text {D}}_{\text {full}}$	${\text {E}}_{\text {full}}$	${\text {F}}_{\text {full}}$	${\text {G}}_{\text {full}}$	${\text {H}}_{\text {full}}$	${\text {I}}_{\text {full}}$	${\text {J}}_{\text {full}}$
Timescale(s)	638	490	425	388	348	549	411	353	308	284

Experiment	${\text {K}}_{\text {full}}$	${\text {L}}_{\text {full}}$	${\text {M}}_{\text {full}}$	${\text {N}}_{\text {full}}$	${\text {O}}_{\text {full}}$	${\text {P}}_{\text {full}}$	${\text {Q}}_{\text {full}}$	${\text {R}}_{\text {full}}$	${\text {S}}_{\text {full}}$	${\text {T}}_{\text {full}}$
Timescale(s)	452	349	306	270	252	411	318	274	250	227

#### Comparing the model with experiments

**Fig. 9:**
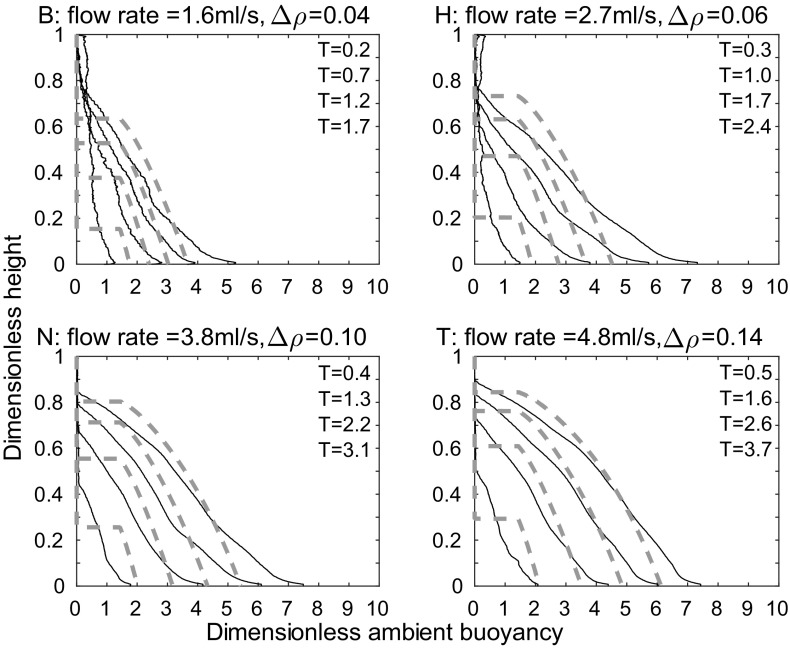
Dimensionless ambient buoyancy profiles for full wall source experiments ${\text {B}}_{\text {full}}$, ${\text {H}}_{\text {full}}$, ${\text {N}}_{\text {full}}$, and ${\text {T}}_{\text {full}}$. *Solid black lines* show filtered experimental results, the *dashed lines* show the model results. Each subplot shows profiles at times 120, 360, 600, and 840 s after starting. The corresponding dimensionless times are listed on each plot

**Fig. 10:**
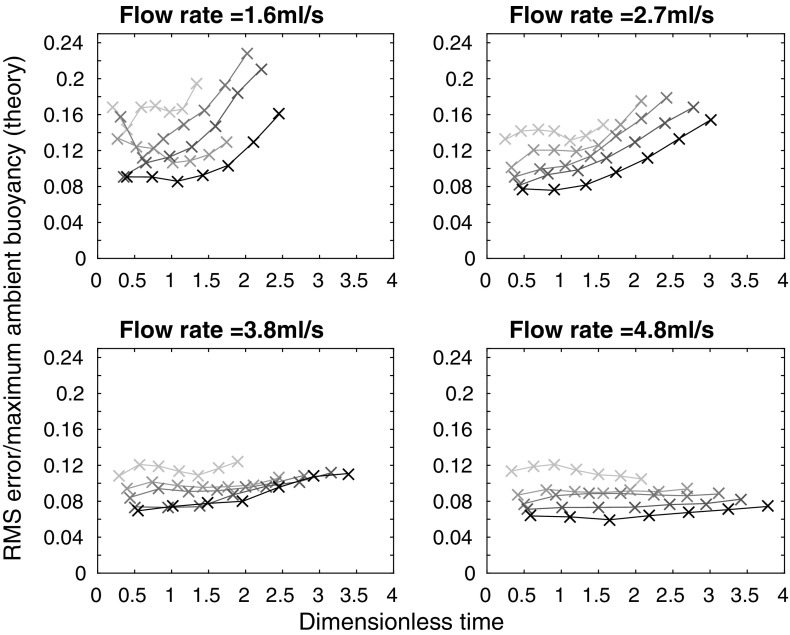
RMS error between theoretical (one-way-entrainment model, with $\alpha =0.018$, chosen to give a low RMS error over all the experiments) and experimental dimensionless ambient buoyancy profiles, divided by the maximum theoretical dimensionless ambient buoyancy, against dimensionless time for each of the 20 full wall source experiments listed in Table 3. Note that the maximum dimensionless times here are approximately 50% of those for the line source shown in Fig. 7. Therefore, comparison with Fig. 7 up to a dimensionless time of approximately 4 is appropriate

Representative experimental results are shown in Fig. 9, along with the one-way-entrainment model of [5], but, as expected, the experimental results and the model disagree. The experimental and theoretical profiles are qualitatively different near $Z=0$ (the base of the tank). The one-way-entrainment model, which is without detrainment, is inadequate for describing our experiments. This is further highlighted by considering the RMS error between the theoretical and experimental dimensionless ambient buoyancy profiles, as shown in Fig. 10. Unlike with the line source, shown in Fig. 7, the RMS error is larger than 8% of the maximum theoretical dimensionless ambient buoyancy, and appears to be increasing with time for all but the largest flow rate. Both the RMS error and visually comparing the shapes of the profiles indicate that the one-way-entrainment model is inadequate for describing the full wall source experiments. We see in the following section that a peeling plume model, which allows for intrusions of plume fluid into the ambient at intermediate heights, captures the experimentally observed change in curvature in the ambient buoyancy profile near the base of the tank.

#### Peeling plume

**Fig. 11:**
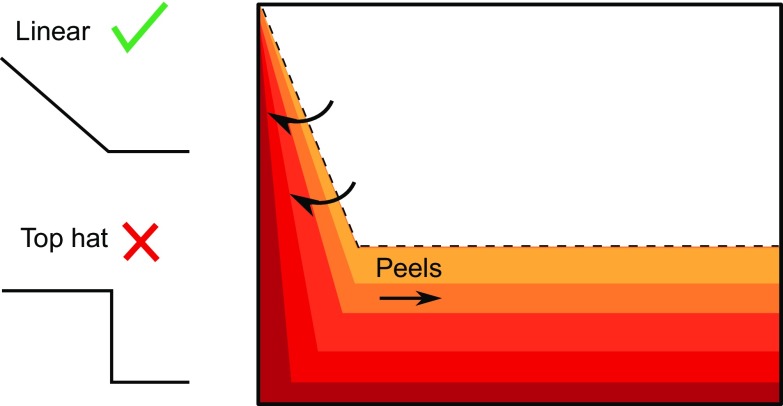
In the peeling plume model, density and vertical velocity vary linearly across the width of the plume, so parts of the plume can peel into the ambient at intermediate heights

The detrainment observed in experiments suggests that some plume fluid reaches its neutral buoyancy height at intermediate heights. The assumption of top hat profiles across the width of the plume rules out this possibility in the model. So, we relax the top hat profile assumption and instead consider linear profiles for vertical velocity and density. Linear profiles allow parts of the plume to peel off at intermediate heights where the density in the plume matches that in the environment, as shown in Fig. 11. This peeling plume model was developed by Hogg et al. [8] to look at the flow of rivers into lakes. In this section we present their model, but extend it from a line source to a full wall source, which has different governing equations (10) from those used by Hogg et al. [8].

To allow the plume to peel at intermediate heights, we now assume, as assumed in [8], that vertical velocity and density vary linearly across the plume:

12 $$\begin{aligned} w = {{\left\{ \begin{array}{ll} w_m(z)(b-x)/b, & x<b, \\ 0, & x>b \end{array}\right. }} \quad {\text {and}} \quad \rho = {{\left\{ \begin{array}{ll} \rho _1+\rho _m(z)(b-x)/b, & x< b,\\ \rho _1, & x>b, \end{array}\right. }} \end{aligned}$$

where *x* is the distance across the plume (from the source wall), *b* is the plume width, $w_{m}(z)$ is the maximum vertical velocity in the plume at height *z*, and $\rho _m(z)$ is the maximum density in the plume at height *z*. Note that we ignore the viscous boundary layer near the wall. On substituting these expressions for vertical velocity and density into (1), we obtain

13 $$q=\frac{w_mb}{2}=Q \left( \alpha ^{2}b_{s_0}H^{4}\right) ^{\frac{1}{3}}, \quad m=\frac{w_m^2b}{3}=M \left( \alpha b_{s_0}^{2}H^{5}\right) ^{\frac{1}{3}}, \quad f=\frac{g\rho _mw_mb}{3\rho _1}=F b_{s_0}H,$$

and Eq. (10) still holds, with boundary conditions (11).

The plume evolves as in the one-way-entrainment model described in Sect. 3.2 until it reaches the first front (the interface between the initial ambient and the stratified part of the ambient). The ambient buoyancy before reaching the first front is $\Delta _a=0$, and there is a similarity solution

14 $$Q=\frac{3}{4}\left( \frac{4}{5}\right) ^{1/3} Z^{4/3}, \quad M=\frac{3}{4}\left( \frac{4}{5}\right) ^{2/3} Z^{5/3}, \quad {\text{and}} \quad F=Z.$$

(This similarity solution is a rescaled version of that found by Cooper and Hunt [5].) By conservation of volume, the first front height $Z_0$ is given by

15 $$\frac{dZ_0}{dT}=-\left. Q\right| _{Z_0,T}.$$

This equation may be integrated, together with $Z_0=1$ at $T=0$, to give the first front height, as found by Cooper and Hunt [5],

16 $$Z_0=\left( 1+\frac{1}{4}\left( \frac{4}{5}\right) ^{1/3}T\right) ^{-3}.$$

After the plume reaches the first front height, we assume that the plume fluid peels and moves to its neutral buoyancy height. The dimensionless buoyancy in the plume varies from zero at the edge of the plume, where plume fluid peels, to the dimensionless maximum buoyancy in the plume,

17 $$\Delta _m=\frac{3F}{2Q}=2\left( \frac{4}{5}\right) ^{-1/3}Z^{-1/3}.$$

Fluid of buoyancy $\Delta _i$ may only begin to peel and enter the stratified part of the tank when the dimensionless maximum buoyancy in the plume at the first front height, $\Delta _m(Z_0(T))$, is equal to $\Delta _i$, i.e. when

18 $$2\left( \frac{4}{5}\right) ^{-1/3}+\frac{T}{2}=\Delta _i.$$

On rearranging this equation, we obtain the time at which fluid of buoyancy $\Delta _i$ begins to peel,

19 $$\begin{aligned} T_i={\left\{ \begin{array}{ll} 2\Delta _i-4\left( \frac{4}{5}\right) ^{-1/3}, & \text {for}\quad \Delta _i>2\left( \frac{4}{5}\right) ^{-1/3}\\ 0, & \text {for} \quad \Delta _i<2\left( \frac{4}{5}\right) ^{-1/3}. \end{array}\right. } \end{aligned}$$

This peeling time is different from that found by Hogg et al. [8], because the full wall source plume governing equations are different from their line source plume governing equations.

Fluid with dimensionless buoyancy $\Delta _i$ is located at a distance

20 $$x_i=b\left( 1-\frac{\Delta _i}{\Delta _m}\right)$$

from the wall. This expression was obtained by rearranging (12). Nondimensionalising using $\alpha H$, the length scale in the *x* direction, the dimensionless plume width is

21 $$\frac{b}{\alpha H}=\frac{1}{\alpha H}\left( \frac{4q^2}{3m}\right) =\frac{4Q^2}{3M}=Z.$$

On substituting this expression for the dimensionless plume width into (20), we find that fluid with dimensionless buoyancy $\Delta _i$ is located at a dimensionless distance

22 $$X_i=\frac{x_i}{\alpha H}=Z\left( 1-\frac{\Delta _i}{\Delta _m}\right)$$

from the wall.

The dimensionless vertical velocity, calculated by using the similarity solutions (14) and definitions of *q*, *m*, *f* in (13), is

23 $$\begin{aligned} W= {\left\{ \begin{array}{ll} \frac{3}{2}\left( \frac{4}{5}\right) ^{1/3}Z^{1/3}\left( 1-\frac{X}{Z}\right) , & X<Z\\ 0, & X>Z. \end{array}\right. } \end{aligned}$$

To find the volume flux $Q_i(\Delta _i,Z)$ in the plume of fluid with buoyancy greater than $\Delta _i$, we integrate the vertical velocity (up to $X_i$),

24 $$Q_i(\Delta _i,Z)=\int _0^{X_i}W \, dX=\frac{3}{4}\left( \frac{4}{5}\right) ^{1/3}Z^{4/3}\left( 1-\frac{1}{4}\left( \frac{4}{5}\right) ^{2/3}Z^{2/3}\Delta _i^2\right) .$$

The depth at which fluid of buoyancy $\Delta _i$ is located is calculated by summing the volume of fluid of each buoyancy arriving at the stratified part of the tank:

25 $$Z_i(\Delta _i,T)=1-\int _{T_i}^T \left. Q_i\right| _{Z_0}\,dT,$$

where $T_i$ is given by (19). Fluid of buoyancy $\Delta _i$ is found to be at height

26 $$\begin{aligned} Z_i= {\left\{ \begin{array}{ll} Z_0+\frac{3}{20}\left( \frac{4}{5}\right) ^{2/3}\Delta _i^2\left( 1-Z_0^{5/3}\right), & \Delta _i<2 \left( \frac{4}{5}\right) ^{-1/3}, \\ 1+Z_0-\frac{3}{20}\left( \frac{4}{5}\right) ^{2/3} Z_0^{5/3} \Delta _i^2 - \frac{4}{\Delta _i^3}, & \Delta _i>2\left( \frac{4}{5}\right) ^{-1/3}. \end{array}\right. } \end{aligned}$$

Since the full wall plume governing equations are different from the line source plume governing equations used by Hogg et al. [8], the heights $Z_i$, at which fluid of buoyancy $\Delta _i$ is found, are also different.

Unlike the line source considered by Hogg et al. [8], with a full wall source, buoyancy is added by the source in the stratified part of the tank. At each height, we account for this extra buoyancy by adding the (as yet unattributed) buoyancy output by the source at that height to $\Delta _i$ from (26), which we now call $\Delta _i^{\text {old}}$. Since the first front passes height *Z* at $T=4\left( \frac{4}{5}\right) ^{-1/3}\left( Z^{-1/3}-1\right)$, the new buoyancy $\Delta _i^{\text {new}}$ is the old buoyancy $\Delta _i^{\text {old}}$, from (26), with a correction for the buoyancy added by the source,

27 $$\Delta _i^{\text {new}}=\Delta _i^{\text {old}}+T-4\left( \frac{4}{5}\right) ^{-1/3}\left( Z_i^{-1/3}-1\right) .$$

The distribution $Z_i$ then gives the ambient stratification.

**Fig. 12:**
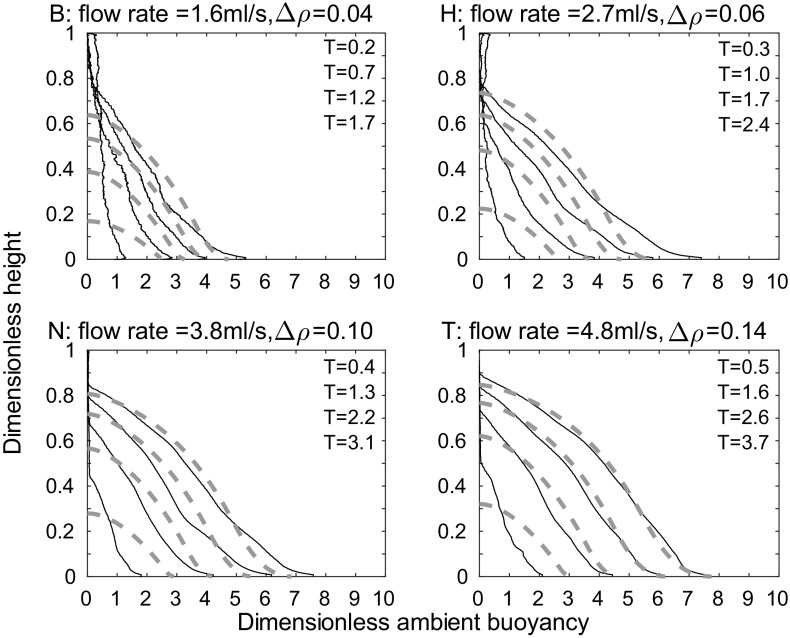
Dimensionless ambient buoyancy profiles for full wall source experiments ${\text {B}}_{\text {full}}$, ${\text {H}}_{\text {full}}$, ${\text {N}}_{\text {full}}$, and ${\text {T}}_{\text {full}}$. *Solid black lines* show filtered experimental results, the *dashed lines* show the peeling plume model results with $\alpha =0.018$. Each subplot shows profiles at times 120, 360, 600, and 840 s after starting. The corresponding dimensionless times are listed on each plot

**Fig. 13:**
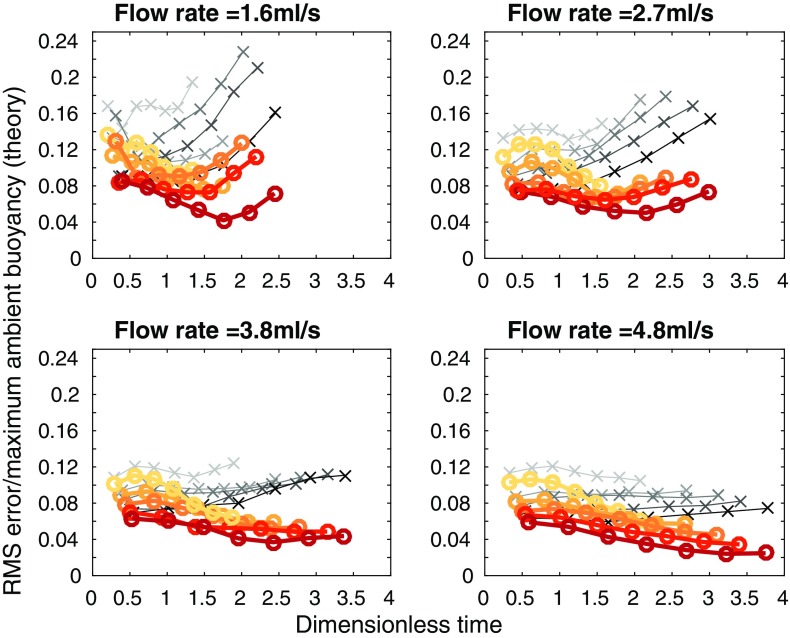
RMS error between theoretical (peeling plume model shown in the* thick orange lines marked* with *circles*, and the one-way-entrainment model shown in the* thin grey lines* *marked* with* crosses*, both models using $\alpha =0.018$, which was chosen to give a low RMS error over all the experiments) and experimental dimensionless ambient buoyancy profiles, divided by the maximum theoretical dimensionless ambient buoyancy, against dimensionless time for each of the 20 full wall source experiments listed in Table 3. Darker colours are experiments with larger source densities

The theoretical profiles given by the peeling plume model, shown in Fig. 12, capture the shape of the ambient buoyancy profiles measured in experiments, whereas the one-way-entrainment model, shown in Fig. 9 does not—the peeling plume model is better at capturing the behaviour near $Z=0$. While, for the full wall source, the agreement is better with the peeling plume model than the one-way-entrainment model, neither model explains which types of source we expect detrainment for, but, if detrainment is present, the peeling plume model may better capture the density profile than the one-way-entrainment model. The agreement is not perfect, however. In particular, in experiments there is generally more dense fluid near $Z=0$ than there is in the model. (This is not always the case at early times, which may be influenced by the crashing of the initial front.) One contribution to this difference is that, in the theoretical model, the extra buoyancy from the source is just added at each height. In practice, however, a plume will form at the lower sources and some of the extra buoyancy will be added to the ambient at a depth nearer to $Z=0$.

We calculate the RMS error between the experimental and the peeling plume theoretical dimensionless ambient buoyancy profiles, shown in Fig. 13. The peeling model is shown by thick orange lines marked with circles, with the one-way-entrainment model shown by thin grey lines marked with crosses. Whilst at the two smaller flow rates, the peeling plume model with $\alpha =0.018$ makes only a little difference to the RMS error (compared with the one-way-entrainment model in Fig. 10), at the two larger flow rates, the RMS error is smaller at late times with the peeling plume model than with the one-way-entrainment model.

**Fig. 14:**
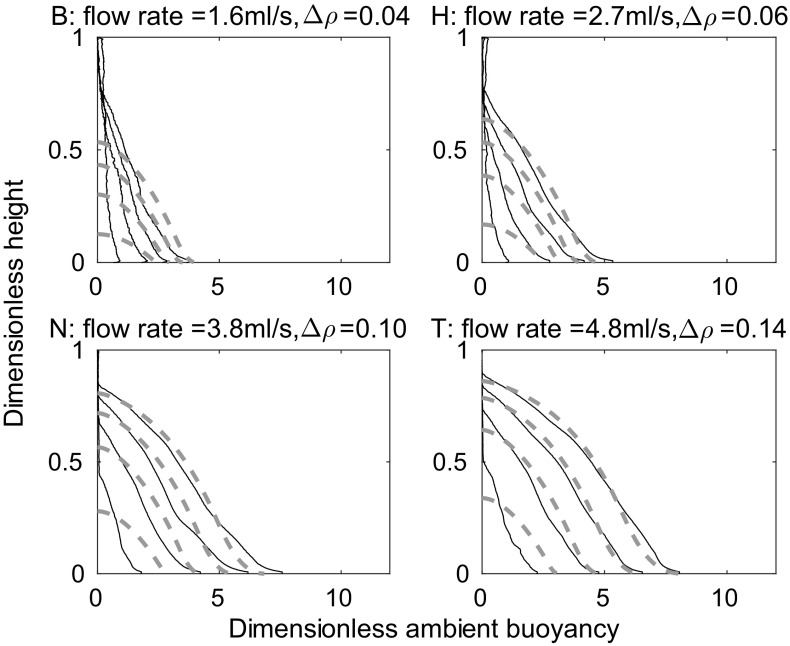
Dimensionless ambient buoyancy profiles for full wall source experiments ${\text {B}}_{\text {full}}$, ${\text {H}}_{\text {full}}$, ${\text {N}}_{\text {full}}$, and ${\text {T}}_{\text {full}}$. *Solid black lines* show filtered experimental results, the *dashed lines* show the peeling plume model results. Each subplot shows profiles at times 120, 360, 600, and 840 s after starting. For a flow rate of 1.6 ml/s, $\alpha =0.011$, for a flow rate of 2.7 ml/s, $\alpha =0.011$, for a flow rate of 3.8 ml/s, $\alpha =0.018$, and for a flow rate n of 4.8 ml/s, $\alpha =0.020$

**Fig. 15:**
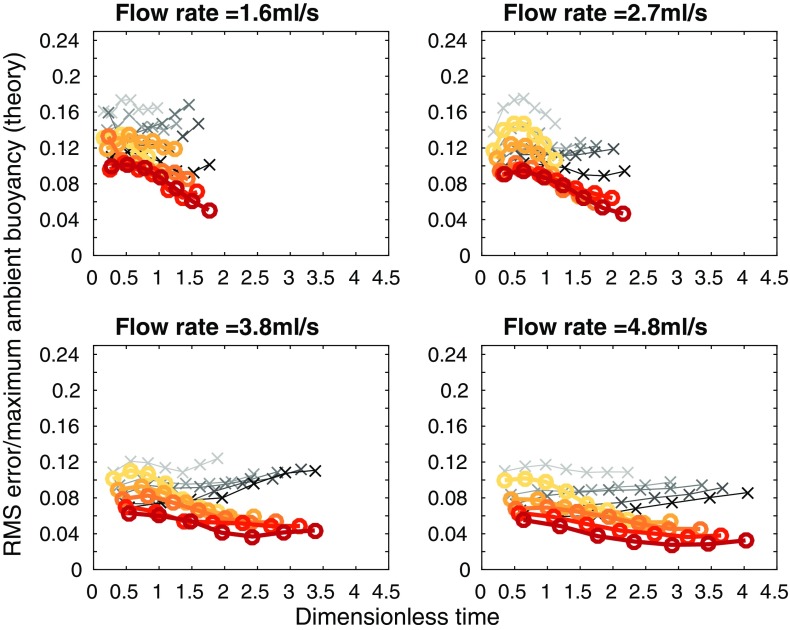
RMS error between theoretical (peeling plume model shown in the* thick orange lines marked* with *circles*, and the one-way-entrainment model shown in the* thin grey lines* *marked* with* crosses*) and experimental dimensionless ambient buoyancy profiles, divided by the maximum theoretical dimensionless ambient buoyancy, against dimensionless time for each of the 20 full wall source experiments listed in Table 3. For a flow rate of 1.6 ml/s, $\alpha =0.011$, for a flow rate of 2.7 ml/s, $\alpha =0.011$, for a flow rate of 3.8 ml/s, $\alpha =0.018$, and for a flow rate of 4.8 ml/s, $\alpha =0.020$. *Darker colours* are experiments with larger source densities

To improve the agreement between the peeling plume model and experiments at smaller flow rates, we can use different values of the entrainment coefficient $\alpha$ for the different flow rates. At smaller flow rates, when there is little peeling, there may still be significant entrainment in the stratified region. This will change the net detrainment, which can be captured in the peeling plume model by selecting different values of $\alpha$ for different flow rates. To select the appropriate $\alpha$, we compare, by considering the RMS error, the first front height predicted by theory (note that this height is the same for both the peeling plume model and the one-way-entrainment model) with that measured in experiments, for a range of values of $\alpha$. In the experiments, there is no sharp first front, rather it is continuous, so we use the height at which the ambient density reaches some value (we somewhat arbitrarily used 0.65 because, looking at the ambient density profiles, this marks out a plausible first front height). We select, for each flow rate, the $\alpha$ that minimises the total RMS error for all experiments at that flow rate. This gives $\alpha =0.011$ for experiments $A_{\text {full}}$ to $E_{\text {full}}$, $\alpha =0.011$ for experiments $F_{\text {full}}$ to $J_{\text {full}}$, $\alpha =0.018$ for experiments $K_{\text {full}}$ to $O_{\text {full}}$, and $\alpha =0.020$ for experiments $P_{\text {full}}$ to $T_{\text {full}}$. Since $\alpha$ is a dimensionless parameter, we expect that the variation with flow rate is via some other dimensionless parameter. Since $\alpha$ varies with flow rate but not with source density, the Reynolds number, which does not involve the source density, may well be the appropriate parameter. The theoretical and experimental profiles with these values of $\alpha$ are shown in Fig. 14. The RMS error between theory and experiment using the values of $\alpha$ given above for both the peeling plume model and the one-way-entrainment model is shown in Fig. 15. Whilst the difference between the peeling plume model and the one-way-entrainment model is small, the RMS error is now decreasing with time, so the model is getting better at larger times, rather than worse, as it was for the two lowest flow rates in Fig.13. At the two larger flow rates, the peeling plume model still gives smaller RMS error at late times than the one-way-entrainment model.

**Fig. 16:**
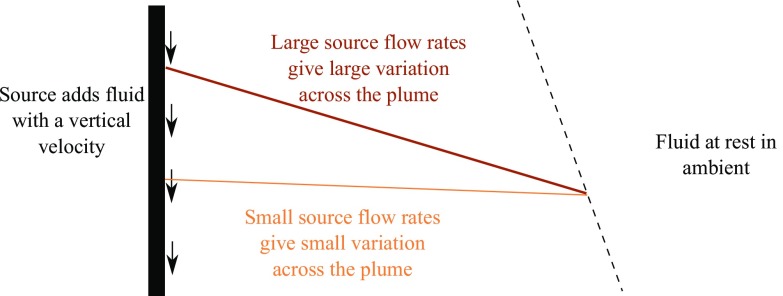
Different source flow rates lead to different variations in vertical velocity across the width of the plume, leading to different amounts of peeling

The agreement between the peeling plume model and experiments is better for larger flow rates than for smaller flow rates because the peeling plume model assumes variation in vertical velocity and in density across the width of the plume. This variation differs with source flow rate, as shown by the schematic in Fig. 16. For large source flow rates, source fluid is added with some velocity near the wall, whilst fluid is at rest in the ambient, so we expect variation in vertical velocity between these two extremes, across the width of the plume. For smaller flow rates, however, there is only a small variation in vertical velocity across the width of the plume, and so there can only be a small amount of peeling. Thus, the peeling plume model shows the best improvement over the one-way-entrainment model at larger flow rates. The RMS error results, together with the better visual agreement between the profiles in Fig. 12, suggest that the peeling plume model is more appropriate than the one-way-entrainment model for describing the dimensionless ambient buoyancy profiles that develop with a full wall source.

## Conclusions

Experimental results have shown that, unlike for a line source, detrainment is important for a full wall source. The existing models, which assume top hat profiles for density and vertical velocity, ruling out the possibility of detrainment, are inadequate. Our experimental results show qualitatively different ambient buoyancy profiles from those predicted by one-way-entrainment models with top hat profiles. Instead, a peeling plume model, with linear profiles for density and vertical velocity across the width of the plume, is appropriate. Extending the peeling plume model of Hogg et al. [8] to our situation gives a better explanation of the experimental results, capturing the shape of the dimensionless ambient buoyancy profiles more accurately. The peeling plume model is an oversimplification of what happens in experiments, however, as several discrete intrusions are observed in experiments (see Fig. 5), rather than intrusions occuring over the entire stratified region. This is potentially due to the discrete sources used in experiments, or alternatively due to the ambient stratification causing discretisation when the plume fluid reaches its neutral buoyancy level, and, as such the model is an oversimplification, but better captures the physics than one-way-entrainment models, which neglect the detrainment that is observed in experiments.

In experiments with a line source, we did not observe detrainment. It is not yet fully understood when detrainment happens, and when it does not. Indeed, it is not yet clear when it is better to approximate the density distribution across the plume as the simplest top hat profile, or when it is more appropriate to approximate this distribution with a linear profile, as we have chosen to do here. Indeed, the existing models, with top hat profiles for density and vertical velocity, appear to agree often with the experimental results. For the line source, our experiments agree with the Worster and Huppert [13] line source model, confirming that our method of approximating a distributed source was appropriate. Through a series of experiments, we have observed that detrainment occurs with a vertically distributed full wall source. With such a source, a peeling plume model, with linear profiles for density and vertical velocity, is appropriate for describing the ambient buoyancy profiles. Detrainment and peeling should be included in models for full wall sources because such models give a qualitatively different ambient profile structure, which better captures profiles measured in experiments, than models with one-way-entrainment only.
